# Novel Water-Based Paints for Composite Materials Used in Electromagnetic Shielding Applications

**DOI:** 10.3390/nano12030487

**Published:** 2022-01-29

**Authors:** Ioan Valentin Tudose, Kyriakos Mouratis, Octavian Narcis Ionescu, Cosmin Romanitan, Cristina Pachiu, Marian Popescu, Volodymyr Khomenko, Oksana Butenko, Oksana Chernysh, George Kenanakis, Viacheslav Z. Barsukov, Mirela Petruta Suchea, Emmanouel Koudoumas

**Affiliations:** 1 Center of Materials Technology and Photonics, School of Engineering, Hellenic Mediterranean University, 71410 Heraklion, Crete, Greece; tudose_valentin@yahoo.com (I.V.T.); kmuratis@hmu.gr (K.M.); 2 Chemistry Department, University of Crete, 70013 Heraklion, Crete, Greece; 3 Institute of Electronic Structure and Laser, Foundation for Research & Technology-Hellas, 71110 Heraklion, Crete, Greece; gkenanak@iesl.forth.gr; 4 National Institute for Research and Development in Microtechnologies (IMT-Bucharest), 023573 Bucharest, Romania; onionescu@gmail.com (O.N.I.); cosmin.romanitan@imt.ro (C.R.); cristina.pachiu@imt.ro (C.P.); fluidproiect@gmail.com (M.P.); 5 Petroleum and Gas University of Ploiesti, 100680 Ploiesti, Romania; 6 Department of Electrochemical Power Engineering and Chemistry, Kyiv National University of Technologies and Design, 01011 Kyiv, Ukraine; v.khomenko@i.ua (V.K.); keeh@knutd.edu.ua (O.B.); chernyshoksana14@gmail.com (O.C.)

**Keywords:** EMI shielding applications, water based paints, conductive paints, multicomponent nanocomposites, graphene nanoplatelets, polyaniline emeraldine (PANI) doped with poly(styrene sulfonic acid) (PSS) or HCl or HBr, poly(3,4-ethylenedioxythiophene) poly(styrene sulfonic acid)—PEDOT:PSS

## Abstract

The development of materials offering electromagnetic interference (EMI) shielding is of significant consideration, since this can help in expanding the lifetime of devices, electromagnetic compatibility, as well as the protection of biological systems. Conductive paints used widely today in electromagnetic interference (EMI) shielding applications are often based on organic solvents that can create safety issues due to the subsequent environment problems. This paper concerned the development of eco-friendly conductive water-based paints for use in EMI-shielding applications. Graphene nanoplatelets, polyaniline emeraldine (PANI) doped with poly(styrene sulfonic acid) (PSS) or HCl or HBr and poly(3,4-ethylenedioxythiophene) poly(styrene sulfonic acid) (PEDOT:PSS) in various ratios were employed in a water base for developing the paints. The target was to develop homogeneous water-based paint-like fluid mixtures easily applied onto surfaces using a paint brush, leading in homogeneous, uniform, opaque layers, draying fast in air at room temperature, and having quite good electrical conductivity that can offer efficient EMI-shielding performance. The results of this parametric trial indicated the optimum compositions leading in paints with optimized properties that can result in uniform, homogeneous, and conductive layers up to a thickness of over 500 μm without deformation and cracking, offering attenuation of up to 60 dBs of incoming GHz electromagnetic radiation. In addition, the structural and morphological characteristics of these paints were studied in detail.

## 1. Introduction

The rapid growth of telecommunications and electronic devices has resulted in increased concerns about electromagnetic interference’s (EMI) impact on environment, living creatures, as well as other electronics. As an example, the emitted electromagnetic radiation from electronic and telecommunication sources can disrupt the functionality of electronics and affect biological systems. Then, the development of materials offering electromagnetic interference (EMI) shielding is of significant consideration, since this can help in expanding the lifetime of devices, electromagnetic compatibility, as well as the protection of biological systems. EMI shielding means the attenuation of incident EM radiation by reflection and absorption by a material, which can act as a barrier against the penetration of the radiation into a system. The reflection loss is tangled with the interaction between the incident wave and mobile charge carriers, as well as withthe impedance discrepancy at the interface of the shielding material. The absorption loss links to the dissipation of electromagnetic wave energy into the shielding materials due to heat loss under the interaction of the electric dipoles in the material and the incident EM radiation. Various nanomaterials have been developed and tested regarding electromagnetic shielding applications, as one can see in some recent and extensive reviews on this subject [1,2,3,4,5,6].

In particular, various forms of carbon, such as graphene, carbon nanotubes, carbon fiber, carbon aerogels, carbon black, activated carbon, and carbon nanoparticles, as well as their hybrid composites, have been extensively developed and studied regarding shielding in the GHz range [1,2,3,4,5,6,7,8,9,10,11]. The microstructures of these carbon materials, the architecture of the shielding coating, and the inclusion of magnetic and dielectric materials in the shield have been found to be significantly beneficial for high EMI-shielding effectiveness. Although the electrical and dielectric properties as well as the shielding effectiveness of polymer/graphene nanoplatelets (GNP) polymer/carbon black (CB) nanocomposites have been intensively investigated [5,9,10,11], there is a lack of studies on multiple component formulations that can result in hybrid composites based on high-structure CB- and GNP-conductive fillers. This approach can be significantly important today since the new 5G technology requires improved EM-shielding properties, especially in the highest bands of frequencies used for wireless applications.

The trials presented in this study were focused on developing homogeneous water-based paint-like fluid mixtures, easily applied onto surfaces using a paint brush, leading in homogeneous, uniform, opaque layers, drying fast in air at room temperature, and having quite good electrical conductivity that can offer efficient EMI-shielding performance. Starting with the synthesis of the required Emeraldine Base, various doped emeraldine salts of high conductivity and compatibility with the other materials used were developed using various types of acids and mixed with graphene nanoplatelets and PEDOT:PSS in water. After initial conductivity tests, the most promising compositions were further used for the preparation of paints. Targeting optimum combination of suitable physical/chemical properties and shielding performance, various ratios of the materials as well as various preparation parameters were tested so that effective shielding paints could be obtained. As a result, paints with optimized properties were developed offering uniform, homogeneous, and conductive layers with a thickness of over 500 μm without deformation and cracking, exhibiting shielding effectiveness of −60 dBs for electromagnetic radiation in the GHz frequency range. The structural and morphological characteristics of these paints were studied in detail.

## 2. Materials and Preparation

The electromagnetic shielding layers were deposited on 16-cm-by-16-cm foam board by brushing paint-like dispersions. Graphene nanoplatelets, polyaniline emeraldine (PANI), doped with poly(styrene sulfonic acid) (PSS) or HCl or HBr and poly(3,4-ethylenedioxythiophene) poly(styrene sulfonic acid) (PEDOT:PSS), were employed in water base for developing the paints. Commercially available graphene nanoplatelets were used, provided by EMFUTUR Technologies Ltd. Castellon, Spain, 5 μm wide, with an average 5 nm thickness, a bulk density of 0.03 to 0.1 g/cc, a carbon content of >99.5 wt%, an oxygen content of <1%, and a residual acid content of <0.5 wt%. Moreover, poly(3,4-ethylenedioxythiophene) poly(styrene sulfonic acid) (PEDOT:PSS) was purchased from Heraeus (Hanau, Germany). Finally, Polyaniline (PANI) was synthesized and doped in our laboratory by a chemical method [12,13], and the electrical properties of the respective salts were examined. Since PANI was synthesized in a single batch, this would have the same dispersion degree for all the samples, the variations in the conductive performance of the samples being rather connected with the presence of the other components. The most conductive water-soluble HCl-, HBr-, and PSS-doped PANI salts were used for the preparation of the paints by the addition of various quantities of graphene nanoplatelets and PEDOT:PSS in water in order to obtain paint-like fluid mixtures. To achieve the strength requirements in practical application, such as peel strength and tensile strength, various preliminary trials were performed and the formulation was successively adjusted until the fluid mixtures became suitable as a paint. The uniformity of the paint thickness was controlled by using a controlled quantity of dry substance in a specific volume. Assuming the paint homogeneity, applying the same quantity of paint on a certain surface the thickness would be approximately the same.

## 3. Characterization Methods

The obtained materials were characterized by scanning electron microscopy (SEM), X-ray diffraction (XRD) and Raman Spectroscopy, and their electrical and shielding properties were evaluated.

SEM characterization was performed using a (FE-SEM) Nova NanoSEM 630 (FEI Company, Hillsboro, OR, USA), equipped with an EDX detector (EDAX TEAM™, Pleasanton, CA, SUA), in order to investigate and understand the formation and the architecture of the obtained nanocomposite materials. All samples were characterized in the high-vacuum mode without any conductive coating. XRD investigations were performed using a Rigaku Ultra high-resolution triple-axis multiple-reflection SmartLab X-ray Diffraction System (Osaka, Japan) in grazing incidence geometry, varying the 2θ from 5° to 50° with a speed of 4°/min. The peak indexing was achieved using ICDD (International Center for Diffraction Data) database. Moreover, Raman analysis was performed using a Witec alpha 300S (Witec Gmbh, Ulm, Germany) system, employing an Nd-YAG laser at 532 nm and confocal Raman microscopy (high-resolution confocal Raman imaging, AFM and SNOM). Adhesion properties were evaluated using the appropriate method of evaluating tape adhesion of a coating system cross-cut Tape Test according to ASTM D 3359 [14]. Finally, the electrical resistance of the nanocomposite samples was conducted by using a FLUKE (Everett, WA, USA) 8846A multi-meter, by using the four-point configuration [15].

The shielding performance of the developed materials was examined in terms of shielding effectiveness; a parameter depends on a number of factors related to both the material and the design use, which can be expressed as:

(1) $$\mathrm{SE}=10\log\left(\frac{P_{i}}{P_{t}}\right)=20\;\log\left|\frac{E_{i}}{E_{t}}\right|$$

where E_i_ and E_t_ are incident and transmitted electric fields, respectively,

The absorbance (A_b_) of the radiation could be calculated by measuring the reflectance (R_e_) and the transmittance of the material and using the formula:

A_b_ = 1 − T_r_ − R_e_

where $R_{e}$ is the reflectance and $T_{r}$ is the transmittance of the material:

$$R_{e}={\left|\frac{E_{r}}{E_{i}}\right|}^{2}={\left|S_{11}\;\mathrm{or}\;S_{22}\right|}^{2}$$

$$T_{r}={\left|\frac{E_{t}}{E_{i}}\right|}^{2}={\left|S_{12}\;\mathrm{or}\;S_{21}\right|}^{2}$$

where $S_{11}$, $S_{12}$, $S_{22}$, and $S_{21}$ are the scattered parameters [16] and could be measured with a Vector Network Analyzer (VNA).

**Figure 1:**
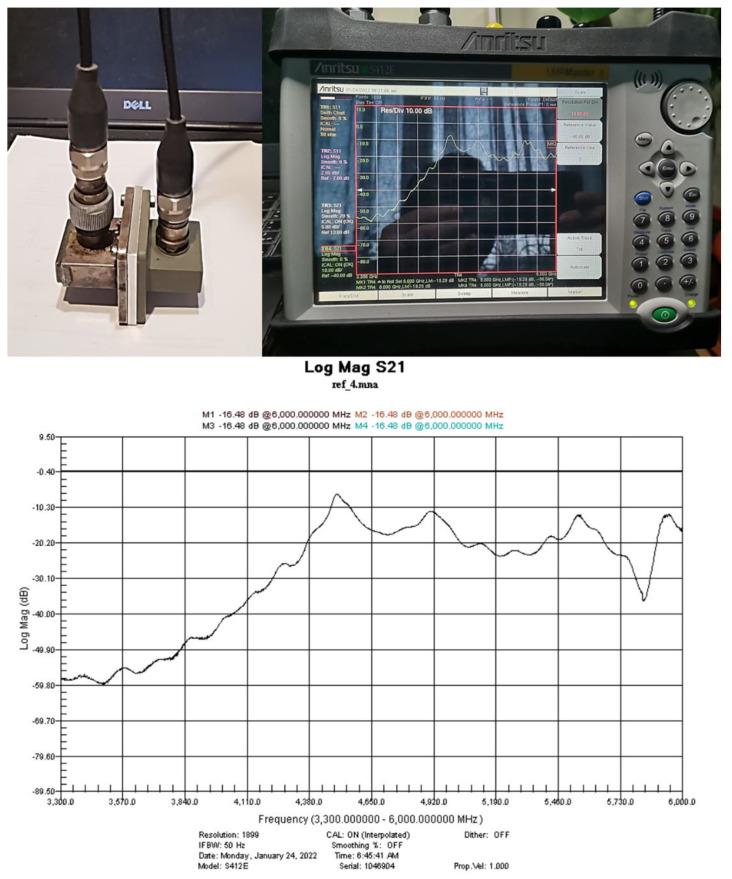
The measuring setup for EMI-shielding efficiency based on the ANRITSU Vector-Network Analyzer.

Two versions of the measuring setup were used for the determination of the shielding performance of the developed paints. The first one (Figure 1) was based on a portable vector-network analyzer Anritsu MS214C: 9 kHz–6 GHz, two Waveguide-to-Coax Adapters, and a diaphragm (holder for sample). The waveguides had a cut-off frequency of 4.3 GHz, resulting in a range of measurement between 4.3 and 6 GHz. Using this setting, the accuracy of measurements was the highest possible, since these were not affected by any interferences.

**Figure 2:**
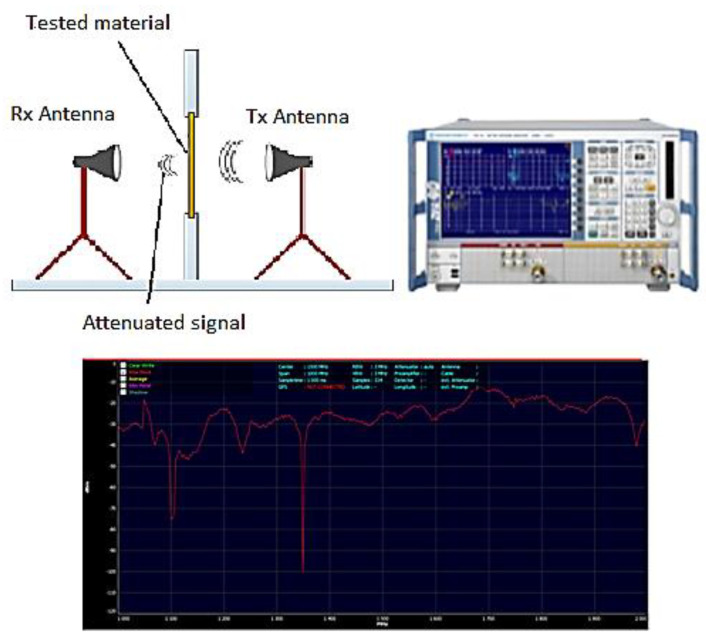
The Rohde & Schwarz measuring setup for EMI-shielding effectiveness.

Extended band measurements were conducted using a vector-network analyzer Rohde & Schwarz ZNB20—shown in Figure 2—with 140 dB dynamic range up to 20 GHz, and antennas for ASTM D4935-10 (modified TEM-cells), that can measure even small samples down to 30 MHz in the far field. The samples were measured with circular radial polarization (all directions, not only horizontal or vertical only), which probably matches the reality.

## 4. Results and Discussion

During the initial trials, pure PANI/graphene/PEDOT:PSS water-based formulations of various compositions were studied, and the resistivity and the shielding efficiency of the best formulations are presented in Table 1.

**Figure 3:**
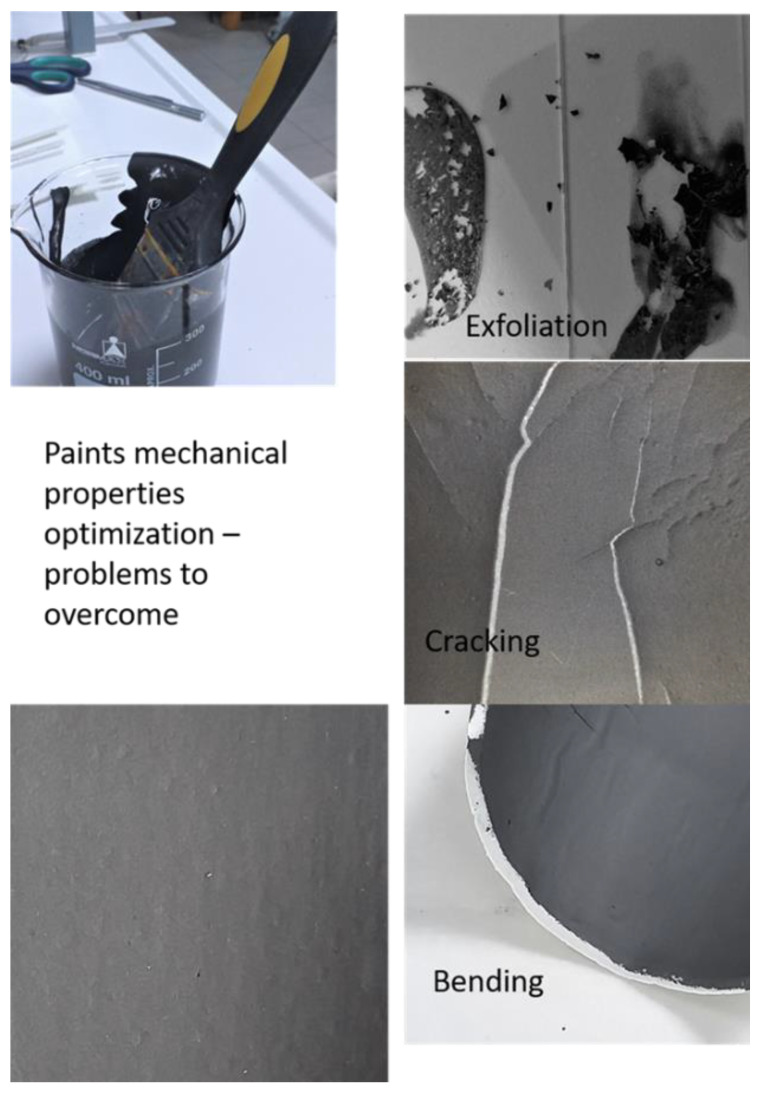
Steps for optimization of the paints.

As a second step, conductive water-soluble doped PANI materials were prepared by a chemical method, so that the most conductive doped PANI could be used to replace pure PANI in the formulations shown in Table 1. As a result of this trial, the most conductive water-soluble doped PANI materials were found to be HCl-, HBr-, and PSS-doped PANI, which were then used for the preparation of the paint-like fluid mixtures by the addition of various quantities of graphene nanoplatelets and PEDOT:PSS in water. The amount of each material to be used was appropriately chosen so that homogeneous paint-like fluid mixtures could be obtained. In each case, the physical/chemical properties as well as the conductivity and the shielding effectiveness were examined, so that paints with optimum properties and performance could be obtained. Except conductivity and the shielding effectiveness, characteristics under investigation were the ease of application of the paints onto surfaces using a paint brush, the quality of the obtained coating regarding homogeneity and uniformity, and the time required for drying in air at room temperature. Then, for the most successful formulations regarding physical/chemical characteristics, more graphene nanoplatelets were added so that the conductivity could be further improved without any downgrading of the basic characteristics. In that respect, step-by-step modification of the composition as well as the preparation parameters were tested. In particular, the effect of stirring type, speed, water content, and temperature variations were studied. Figure 3 presents some of the steps taken for the optimization of the paint. The conclusion of this study was that addition of 5 g of graphene nanoplatelets in 200 mL water-based paint-like doped PANI/graphene/PDOT:PSS preserved/improved the paint physical properties without canceling any of the coatings’ physical-chemical properties and led to uniform, homogeneous layers up to a thickness of over 500 μm without deformation and cracking.

**Figure 4:**
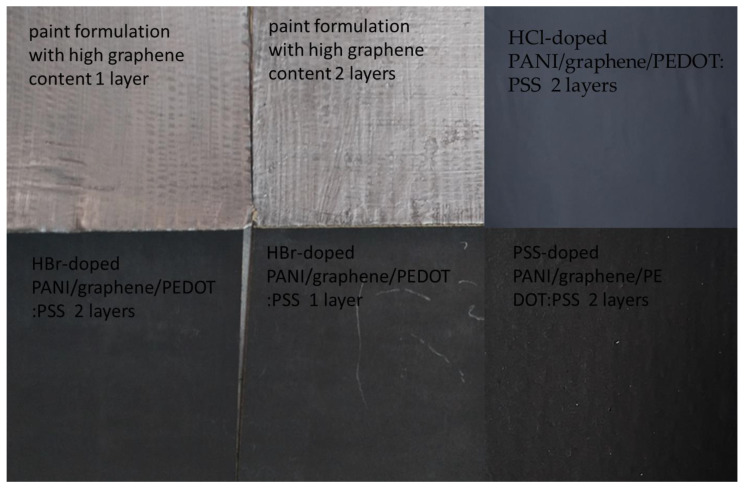
Examples of good-quality coatings onto large (16 × 16 cm^2^) paper boards used for EMI-shielding properties characterization.

After the optimization of the formulation, excellent-quality coatings were obtained. Figure 4 shows some examples of large 16 × 16 cm^2^ paper boards good-quality coatings used for EMI-shielding properties characterization. Then, coatings based on these optimum formulations were further studied regarding their morphology and structure.

**Figure 5:**
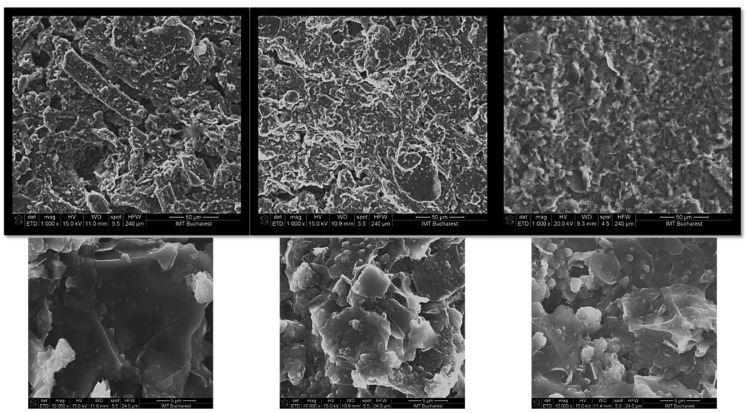
Images of SEM characterization of HCl-doped PANI/graphene/PEDOT:PSS water-based coatings, with one, two, and three layers, at two magnifications (scales of 50 μm and 5 μm).

A detailed examination of the paints at microscopic scale was performed by using SEM. Examples of SEM characterization images of materials obtained from optimized HCl-doped PANI/graphene/PEDOT:PSS water-based formulations with different numbers of applied layers (one, two, and three) are presented in Figure 5. For the case of HBr- and PSS-doped PANI/graphene/PEDOT:PSS water-based formulations, SEM characterization showed similar surface main characteristics as well as similar trends with an increasing number of layers.

As one can observe in Figure 5, the application of a single layer led to an inhomogeneous rough surface, with a non-uniform thickness. Increasing the number of applied layers obviously led to a more compact, homogeneous material with a smoother surface. This behavior at the microscopic level seemed to directly affect the electrical properties of the coatings, as will be presented later on.

**Figure 6:**
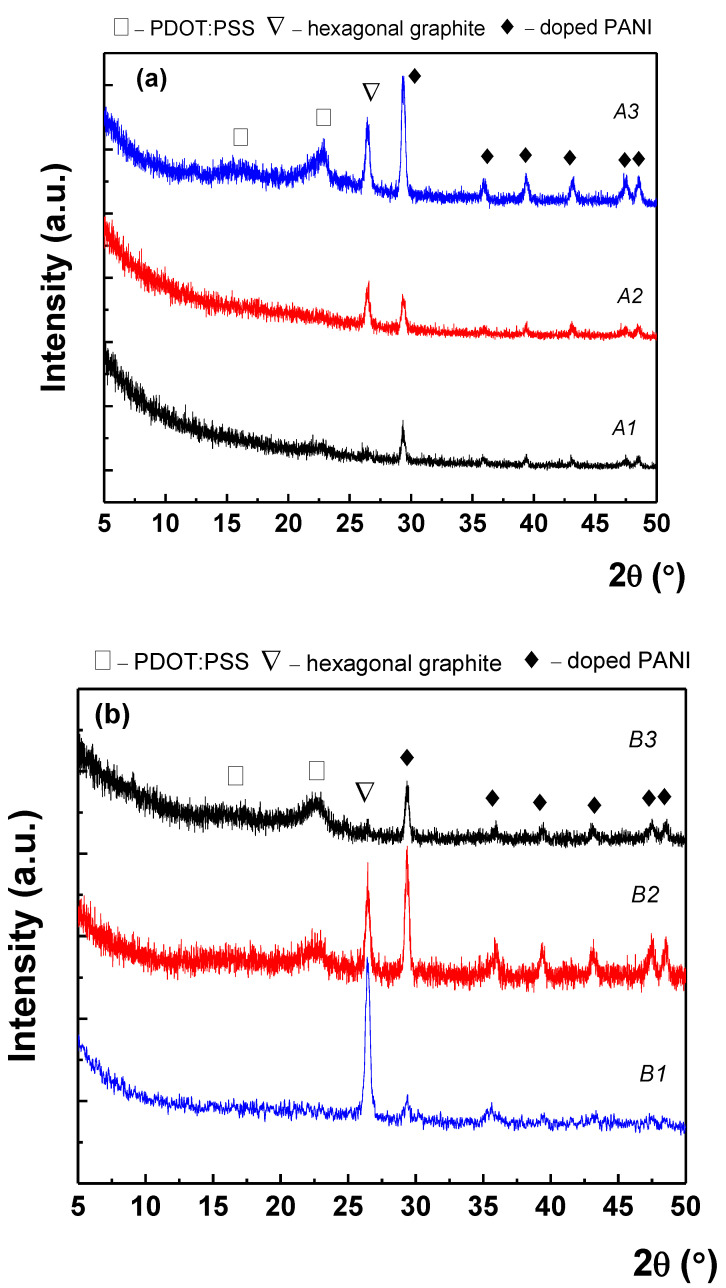

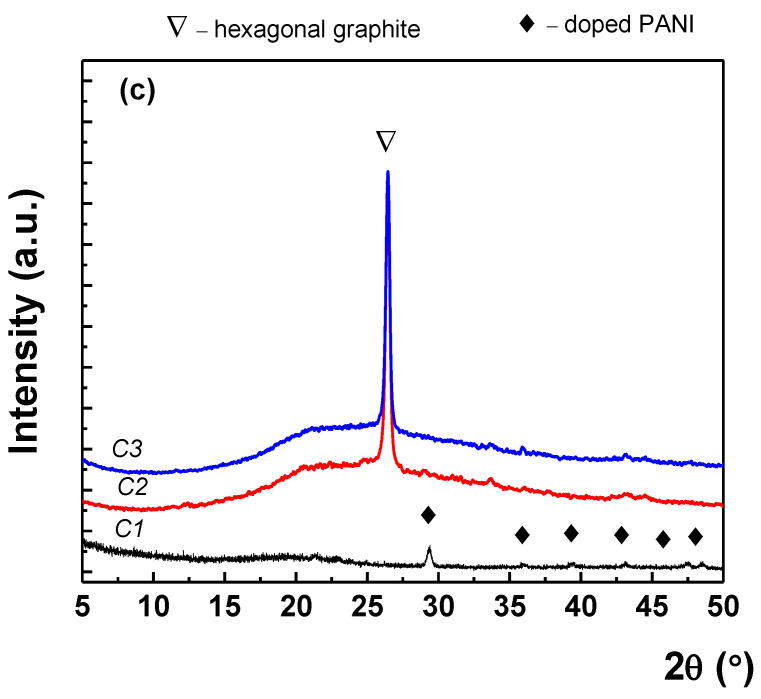
XRD profiles of (**a**) HCl, (**b**) HBr, and (**c**) PSS-doped PANI/graphene/PEDOT:PSS water-based formulation with one, two, and three layers (black, red, and blue, respectively).

X-ray diffraction was employed to determine the structural properties of the paints and to evaluate the effect of the number of applied layers in the nanocomposite microstructure. As can be observed in Figure 6a, each X-ray diffraction pattern exhibited multiple diffraction features/peaks. For example, in the case of A1, only weak diffraction features could be observed, while, with increasing the number of the layers, the diffraction peaks became sharper and, moreover, a new diffraction peak occurred at 26.4°, which could be assigned as (002) reflection of the hexagonal graphite according to the ICDD database (card no. 041-1487). Using Bragg’s law [17], the distance between adjacent layers can be calculated to be 0.34 nm. The diffraction features located at 15.9° and 22.7° were assigned as PEDOT:PSS, which typically exhibits such a spectrum in this region [18,19]. The other peaks could be the contribution of doped PANI according to the synthesis processes, but a proper indexing of the peaks with Miller indices was not possible using ICDD database or previous published papers in the field. Finally, in the case of A3, the relative intensity ratio (RIR) value of graphite becomes 32%, this value evaluated based on the integral area of the diffraction peaks fitted with the Voigt function [20]. The respective X-ray diffraction patterns of the HBr-doped PANI/graphene/PEDOT:PSS water-based formulation are presented in Figure 6b. The use of HBr as dopant led to a different structuring of the EMI-shielding coating, since the increasing of the applied layers leads to a drastic decreasing of the intensity of the graphene diffraction peak. In particular, in the case of B1, the graphene was predominant, while in the case of B3, the XRD pattern indicated only the presence of PEDOT:PSS and doped PANI_._

Finally, for the PSS-doped PANI/graphene/PEDOT:PSS water-based formulations, while in the case of one layer the diffractogram was dominated by the doped PANI pure phase, increasing the number of applied layers resulted in diffractograms dominated by the presence of graphene. In this context, it seems that the type of doping of PANI and the number of applied layers can control the structuring of the paint. Finally, it should be mentioned that the good crystal quality of the doped PANI in the composites under study was proven by the mean crystallite size, which was found to be around 17 nm, based on Scherrer’s equation and Rietveld refinement, respectively.

**Figure 7:**
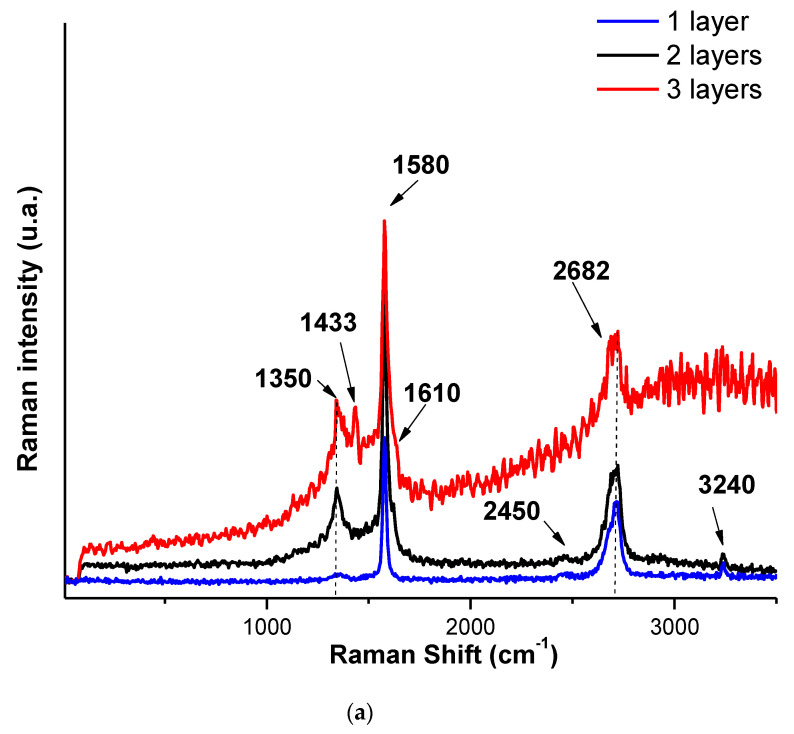

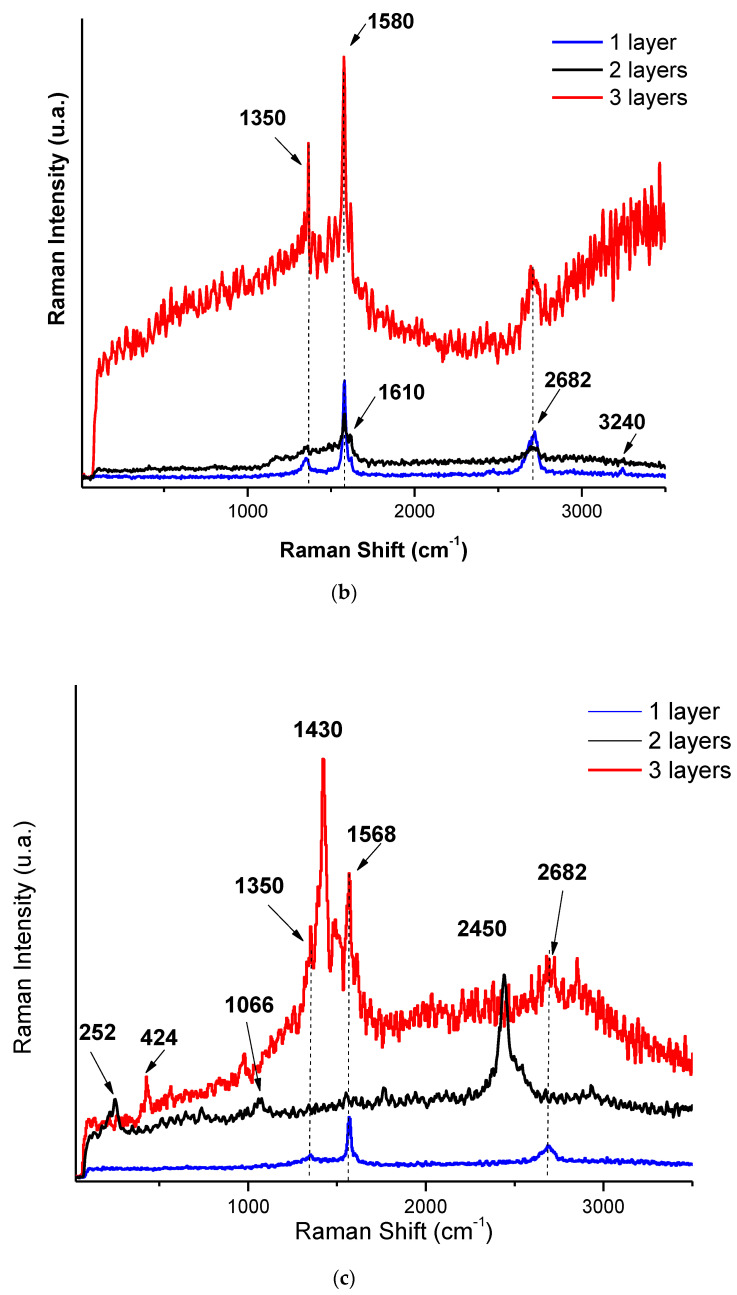
Raman spectra of (**a**) HCl-, (**b**) HBr-, and (**c**) PSS-doped PANI/graphene/PEDOT:PSS water-based formulations with one, two, and three layers.

The interactions between the HCl- or the HBr-doped PANI with graphene and PEDOT:PSS is expected to determine the intensity or the position of the Raman peaks. Raman spectra of the materials are presented in Figure 7, where the following bands can be seen:

For the case of HCl- and HBr-doped PANI/graphene/PEDOT:PSS composites, the G band of graphene, corresponding to the vibration of sp2-bonded carbon atoms, the C−C bonds in the graphitic structure were found at about 1580 cm^−1^, presenting a shift to lower wavenumbers with respect to the position usually reported (near 1585 cm^−1^). In addition, the D band, indicating the defects or disorders present in structures in carbon-based materials, was found at about 1350 cm^−^^1^, having a shift to longer wavenumber with respect to the position usually reported (near 1340 cm^−1^). Moreover, the G band was split into two peaks, the G-peak and the D’-peak (1610 cm^−1^), due of the random distribution of the composite components in relation with the graphene surface. The sp2 carbon-based materials also exhibited a strong peak in the range 2500–2800 cm^−1^ [21]. Finally, the 2D-band was found at about 2682 cm^−^^1^, and it is related to the stacking order of graphene layers. An additional peak could be seen due to coupling of various bands such as D + G and D + D’, which appear at higher regions (approaching or around 2450 cm^−1^ and 3240 cm^−1^).

The peak C=C symmetric stretching characteristic of PEDOT:PSS can be observed at about 1433 cm^−1^ in the Raman spectra of samples based on HCl-doped PANI, a band slightly shifted with respect to pure PEDOT:PSS films (1445 cm^−1^) [22]. This behavior confirms the π-π interaction of aromatic structures of PEDOT:PSS and hence more conductive configuration of graphene [22]. In the Raman spectrum of HBr-doped PANI/graphene/PEDOT:PSS, the same behavior appeared, but the intensity of the observed PEDOT:PSS peak was much lower. For the case of PSS-doped PANI, the presence of PSS in the respective composite was verified to be the low frequencies located at 252 cm^−1^, 424 cm^−1^, and 1065 cm^−1^ respectively [4,23]. No clear contribution of PANI to the spectra was observed, suggesting that it may be amorphous or bellow detection limits. These observations are in a good agreement with the XRD observations of the presence of crystalline graphene and PEDOT:PSS in the composites.

Particularly, as indicated with the results shown earlier, the number of layers seems to affect the structuring of the materials since:

(a) As shown in Figure 6a, in the material with the HCl-doped PANI, one layer of paint resulted in clearly visible peaks of doped PANI, with a hint of the (002) hexagonal graphite peak and of the 22.7° PEDOT:PSS peak. For two layers, the doped PANI peaks became sharper and the (002) hexagonal graphite peak became clear, while, for three layers, the previously observed peaks became even sharper and a second peak of PEDOT:PSS became visible. This can be associated with improved materials crystallinity as the number of layers increases, in agreement with the SEM observations. A similar conclusion can be drawn from the Raman spectra, their evolution with the number of layers being consistent with the XRD observations.
(b) In the case of the material with HBr-doped PANI (Figure 6b), a very sharp (002) hexagonal graphite peak appeared with weak peaks of doped PANI for one layer. Then, two layers showed smaller (002) hexagonal graphite peaks and clearly improved peaks of doped PANI with a hint of the 22.7° peak of PEDOT:PSS. Finally, three layers showed the presence of all components, but their intensity became very small, the (002) hexagonal graphite peak almost disappearing. This behavior can be associated to the randomization of the graphene nanoplatelets distribution within the material and an overall order degradation. Raman spectra showed only peaks associated to graphene were present, their intensity decreasing as the number of layers increased.
(c) Finally, for the case of the material with PSS-doped PANI, one layer presented only weak peaks of PSS, while, as the number of layers increased, the hexagonal graphene peak appeared quite sharp, and no peak for PEDOT:PSS was observed. In Raman spectroscopy study, the one-layer spectrum was dominated by the G band of graphene and the 2D-band contributions, while, the two-layer spectrum was dominated by the apparition of the additional peak due to coupling of D + G bands, also presenting the contribution of PSS. Finally, in the case of three layers, the C=C symmetric stretching characteristic peak of PEDOT:PSS became dominant, while the graphene peaks were still present together with the contribution of PSS.

Based on these observations, it seems that the number of layers tended to strongly affect the material structure; therefore, further studies are needed in order to clarify this unexpected behavior. No previously reported studies were found in the literature regarding this aspect.

Regarding the results of the measurements of the electrical resistance, these are presented in Table 2. As can be observed, formulations based on HCl-doped PANI exhibited the lowest resistance, followed by the ones based on HBr- and PSS-doped PANI. As expected, the number of layers of paint affected the resistivity of the coatings, a behavior that can be due to two factors: the thickness and the microscopic morphology. Regarding the thickness, following the formula of resistance $R=\frac{\rho \cdot L}{S}$ (where ρ=resistivity, *L* = length of the sample, and *S* is the area of its cross section). Taking into account that both ρ and *L* were kept constant, then, as the thickness of the paint increases, the resistance will decrease accordingly.

Regarding the microscopic surface morphology, as was presented in the discussion of the SEM characterization, increasing the number of applied layers led to a more compact, homogeneous material with smoother surface, a behavior that can affect the resistivity of the coating.

**Figure 8:**
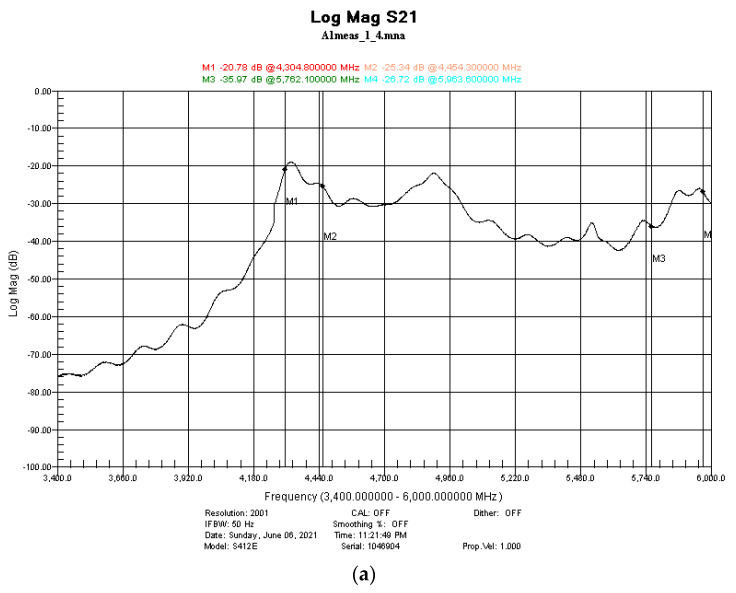

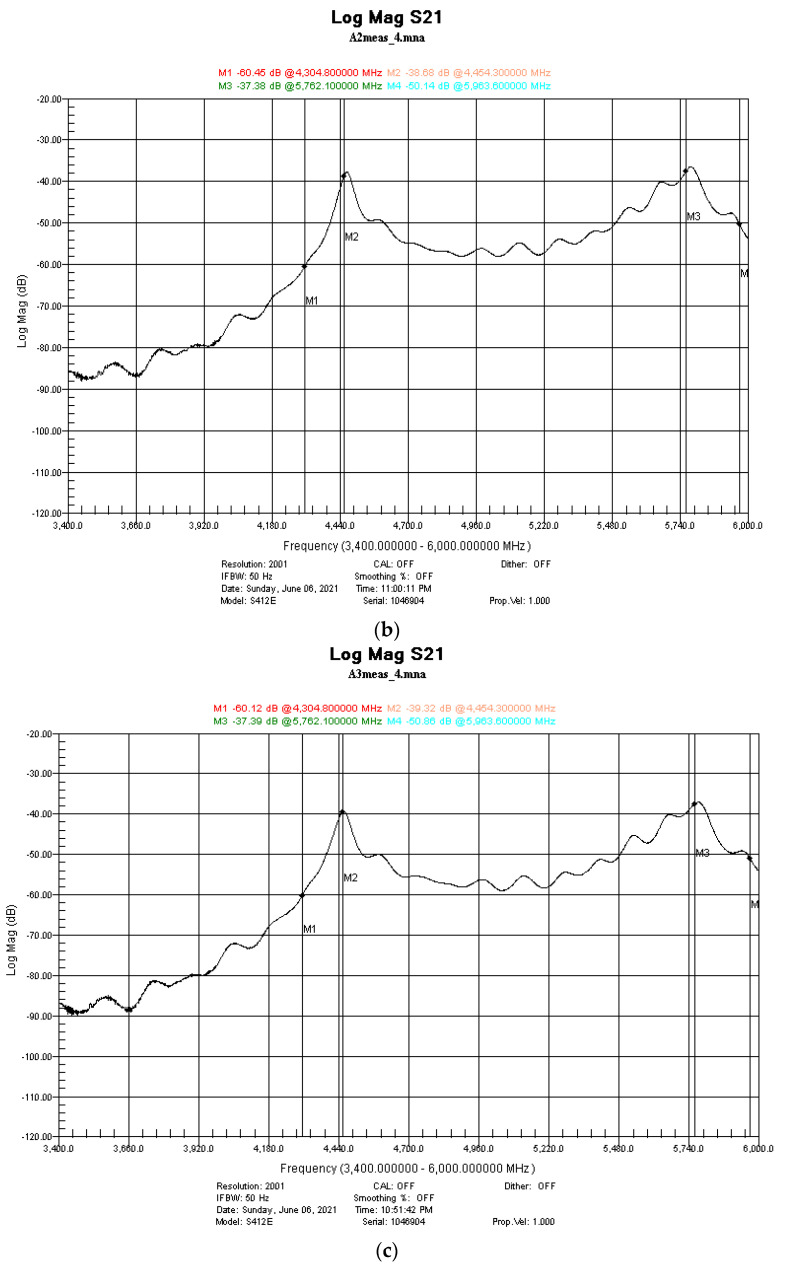
Examples of electromagnetic shielding effectivity of (**a**) one layer, (**b**) two layers, and (**c**) three layers of HCl-doped PANI/graphene/PEDOT:PSS water-based formulation with different numbers of applied layers.

High-precision measurements were conducted in the frequency range 4.3 up to 6 GHz by using the setting presented in Figure 1. After the system calibration using an empty holder, samples with 1, 2, and 3 layers of paint were measured and the results are presented in Figure 8.

**Figure 9:**
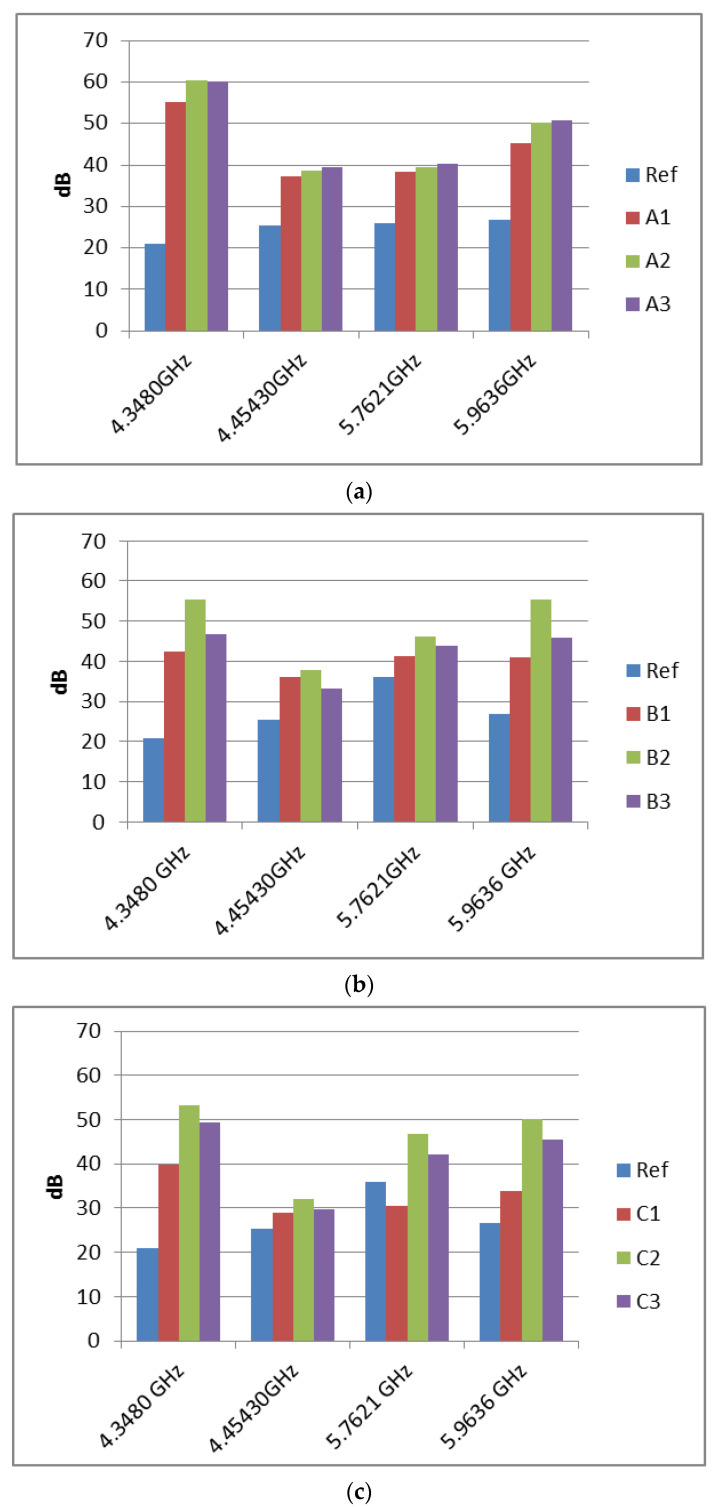
Comparison of the EMI absorption (parameter S_12_) for the three (**a**) HCl and (**b**) HBr and (**c**) PSS-doped PANI/graphene/PEDOT:PSS water-based formulation, using one, two, and three layers from each type.

Moreover, extended measurements were conducted on the same samples, where four markers were established at 4.348, 4.454, 5.762, and 5.963 GHz, in order to have a more clear idea of the attenuation amplitude. The results are presented in Figure 9.

**Figure 10:**
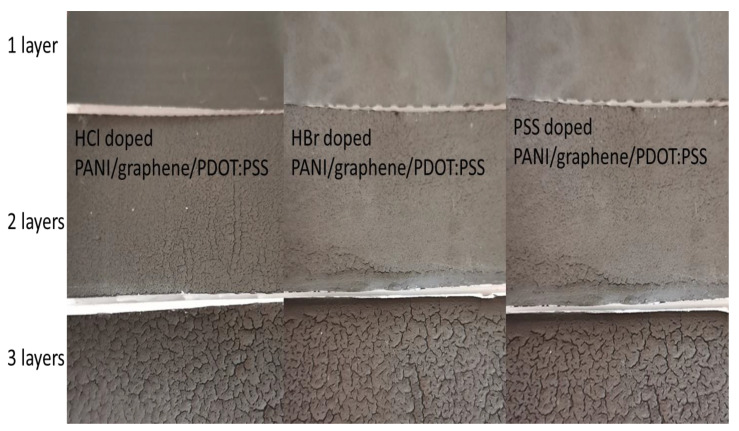
Photos of coatings made with doped PANI/graphene/PEDOT:PSS water-based formulations of one, two, and three layers.

The measurements conducted showed that the shielding behavior was not constant in the studied band, since different frequencies were attenuated differently. It could also be observed that the third layer of all coatings did not significantly improve the shielding performance. This behavior can be attributed to the macroscopic morphology. Figure 10 shows photographs of the doped PANI/graphene/PEDOT:PSS water-based formulation with one, two, and three layers. As can be seen, the third layer presented macro-cracks (not observable by microscopic techniques) for all three types of paints studied, cracks that can lead in less-effective shielding performance. Then, the two-layer coating presented maximum shielding performance since it combined a uniform surface at a macroscopic level with lower resistivity.

Finally, the adherence of the improved HCl- and HBr-doped PANI/graphene/PEDOT:PSS water-based formulation paints to various substrates was measured using method of evaluating tape adhesion of a coating system cross-cut Tape Test according to ASTM D 3359 and is presented in Supplementary file S1. It was found that paints A and B had good adhesion at 4B-5B for the following surfaces: glass, caprolon, cardboard, and tile. Paint A showed the best adhesion to glass and paint B was better for tiles. In contrast, both A and B paints did not present suitable adhesion to plastics such as plexiglass, PET, PBT, and polyacetal. Paints A and B showed a light peeling of a thin top coat from concrete.

Regarding the overall performance of the paints that were studied, these were found to exhibit not only excellent physical/chemical properties, but also to offer very effective electromagnetic shielding in the GHz frequency range, better than that determined in many reports in the literature [1,2,3,4,5,6,7,8,9,10,11,12]. Moreover, the paints can be easily applied to produce homogeneous, uniform, opaque layers, draying fast in air at room temperature, and being adherent on many types of surfaces. Therefore, they are quite suitable for real-life applications. However, further investigations are required for a better understanding of the relative contribution of the GNP, doped PANI and PEDOT:PSS in the structuring, the morphology, and the performance of the composite material, so that further improvement of the shielding efficiency can be obtained.

## 5. Conclusions

Results were presented related to homogeneous water-based paint-like fluid mixtures, offering efficient electromagnetic shielding. These fluid mixtures could be easily applied onto surfaces using a paint brush, leading in homogeneous, uniform layers, drying fast in air at room temperature, having very good adherence on many surfaces, and presenting quite good electrical conductivity, resulting in efficient shielding performance. The fluid mixtures were based on emeraldine PANI doped with acids, showing high conductivity and compatibility with the other materials employed, graphene nanoplatelets and PEDOT:PSS. Various ratios of the materials as well as various preparation parameters were tested so that effective shielding paints could be obtained, offering an optimum combination of physical/chemical properties and effective shielding performance. Paints with optimized properties were found to offer uniform, homogeneous, and conductive layers with a thickness of over 500 μm without deformation and cracking, exhibiting shielding effectiveness up to −60 dBs for electromagnetic radiation in the GHz frequency range. The structural and morphological characteristics of these paints were also studied in detail. It was found that increasing the number of applied layers resulted in a more compact, homogeneous material with smoother surfaces at the microscopic level. Moreover, it was observed that the type of doping of PANI and the number of applied layers could control the structuring of the paints. Further studies regarding the composites’ properties and their correlation with the EMI-shielding effectiveness are ongoing.

## Figures and Tables

**Table 1:** Conductivity and average shielding efficiency for PANI/graphene/PEDOT:PSS coatings of various compositions.

A/A	Sample Composition	Surface Resistivity [Ohm]	Average Shielding Efficiency 1–2 Ghz
1	5 g Graphene, 2 g PANI, 20 mL PEDOT	6.2 × 10^3^	22
2	3 g Graphene, 4 g PANI, 40 mL PEDOT	1.9 × 10^2^	24
3	5 g Graphene, 1 g PANI, 30 mL PEDOT	5.2 × 10^2^	25

**Table 2:** Resistance of paints under investigation.

Number of Layers	HCl Doped PANI Formulations [Ohm]	HBr-Doped PANI Formulations [Ohm]	PSS Doped PANI Formulations [Ohm]
1	1.9 × 10^2^	5.2 × 10^2^	6.2 × 10^3^
2	7.6 × 10^1^	3.2 × 10^2^	3.0 × 10^3^
3	4.1 × 10^1^	1.8 × 10^2^	2.0 × 10^3^

## Data Availability

The raw and processed data required to reproduce these findings cannot be shared at this time due to technical or time limitations. The raw and processed data will be provided upon reasonable request to anyone interested anytime until the technical problems are solved.

## Supplementary Materials

The following supporting information can be downloaded at: https://www.mdpi.com/article/10.3390/nano12030487/s1. The following supporting information regarding “Testing the adherence of the improved HCl- and HBr-doped PANI/graphene/PEDOT:PSS water-based formulation paints to various substrates” can be downloaded at: https://www.mdpi.com/article/10.3390/nano12030487/s1, Supplementary information File S1.
