# Supplementary material for: Novel Water-Based Paints for Composite Materials Used in Electromagnetic Shielding Applications
Source: Nanomaterials (Basel). 2022 Jan 29;12(3):487. doi: 10.3390/nano12030487 (PMC8838246; doi:10.3390/nano12030487)
Supplement: Supplementary file 1 [file nanomaterials-12-00487-s001.zip › nanomaterials-1553536-supplementary.pdf]

nanomaterials

Supplementary Information

Novel Water-Based Paints for Composite Materials Used in Electromagnetic Shielding Applications

Ioan Valentin Tudose [1,2,3], Kyriakos Mouratis [1], Octavian Narcis Ionescu [4,5], Cosmin Romanitan [4], Cristina Pachiu [4], Marian Popescu [4], Volodymyr Khomenko [6], Oksana Butenko [6], Oksana Chernysh [6], George Kenanakis [3], Viacheslav Z. Barsukov [6,*], Mirela Petruta Suchea [1,4,*] and Emmanouel Koudoumas [1,*]

[1] Center of Materials Technology and Photonics, School of Engineering, Hellenic Mediterranean University, 71410 Heraklion, Crete, Greece; tudose_valentin@yahoo.com (I.V.T.); kmuratis@hmu.gr (K.M.)
[2] Chemistry Department, University of Crete, 70013 Heraklion, Crete, Greece
[3] Institute of Electronic Structure and Laser, Foundation for Research & Technology-Hellas, 71110 Heraklion, Crete, Greece; gkenanak@iesl.forth.gr
[4] National Institute for Research and Development in Microtechnologies (IMT-Bucharest), 023573 Bucharest, Romania; onionescu@gmail.com (O.N.I.); cosmin.romanitan@imt.ro (C.R.); cristina.pachiu@imt.ro (C.P.); fluidproiect@gmail.com (M.P.)
[5] Petroleum and Gas University of Ploiesti, 100680 Ploiesti, Romania
[6] Department of Electrochemical Power Engineering and Chemistry, Kyiv National University of Technologies and Design, 01011 Kyiv, Ukraine; v.khomenko@i.ua (V.K.); keeh@knutd.edu.ua (O.B.); chernyshoksana14@gmail.com (O.C.)
* Correspondence: v-barsukov@i.ua (V.Z.B.); mirasuchea@hmu.gr or mira.suchea@imt.ro (M.P.S.); koudoumas@hmu.gr (E.K.)

Citation: Tudose, I.V.; Mouratis, K.; Ionescu, O.N.; Romanitan, C.; Pachiu, C.; Popescu, M.; Khomenko, V.H; Butenko, O.; Chernysh, O.; Kenanakis, G.; et al. Novel Water-Based Paints for Composite Materials Used in Electromagnetic Shielding Applications. *Nanomaterials* **2022**, *12*, 487. https://doi.org/10.3390/nano12030487

Academic Editor: Jin Suk Chung

Received: 28 December 2021
Accepted: 21 January 2022
Published: 29 January 2022

Publisher's Note: MDPI stays neutral with regard to jurisdictional claims in published maps and institutional affiliations.

Copyright: © 2022 by the authors. Licensee MDPI, Basel, Switzerland. This article is an open access article distributed under the terms and conditions of the Creative Commons Attribution (CC BY) license (https://creativecommons.org/licenses/by/4.0/).

Table S1. The adherence of the best the improved HCl (A) and HBr (B) doped PANI/graphene/PDOT:PSS water based formulation paints to various substrates measured using method of evaluating tape adhesion of a coating system cross-cut Tape Test according to ASTM D 3359.

**Substrate material+ Version of paint**	**Applied paint**	**After scraping**	**Macroshot**	**After tearing off the tape**	**Residuum at the Scotch tape**	**The standart ATSM D 3359 – 02***	
**Glass + A**	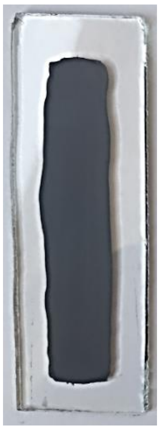	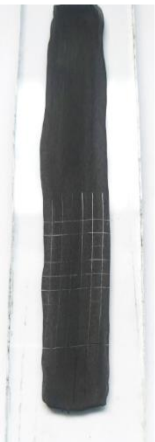	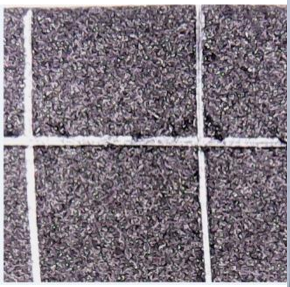	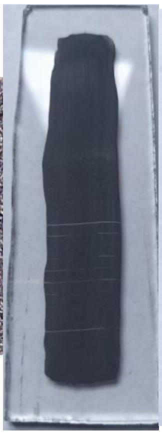	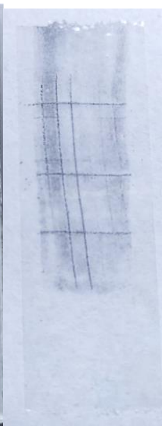	5B	0 % None
Glass +**B**	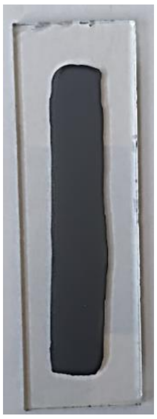	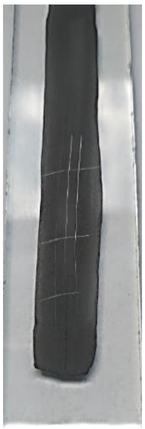	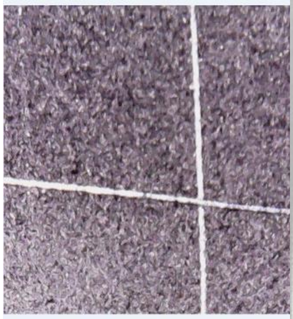	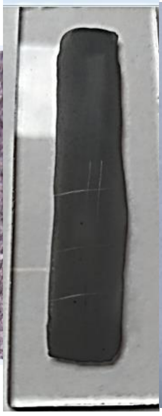	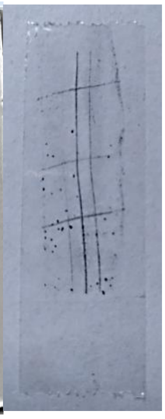	4B	Less than 5 %
Glass +**Base**	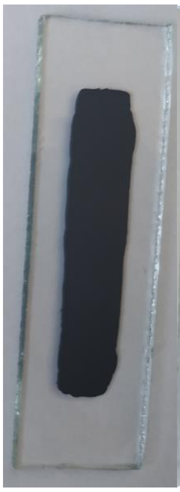	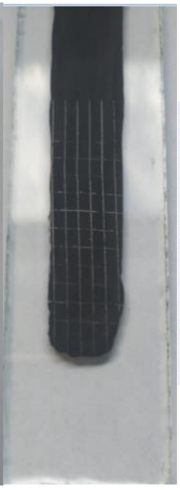	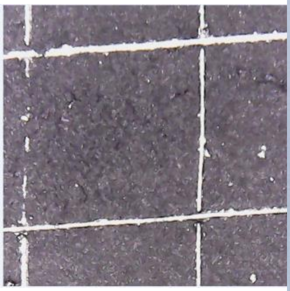	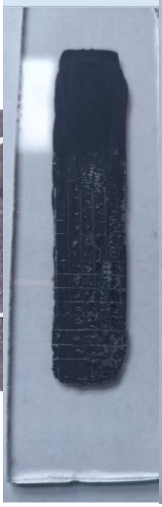	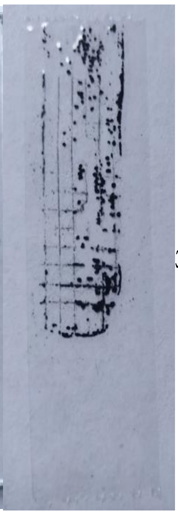	3B	5-15 %

Plexiglass+ **A**	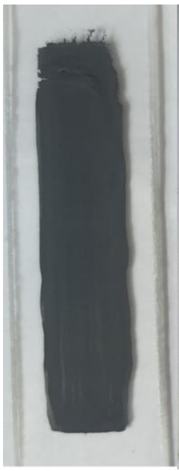	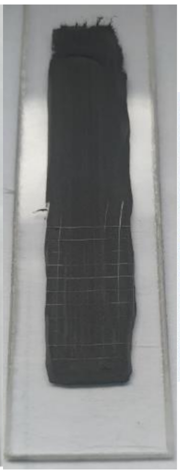	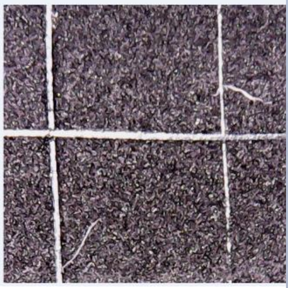	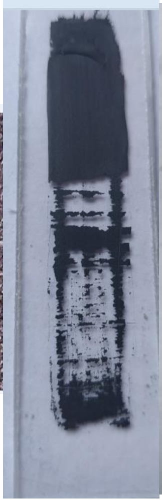	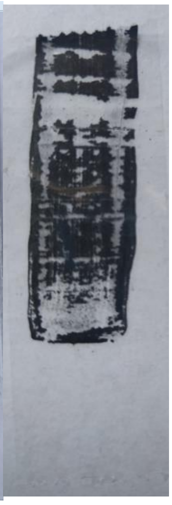	0B	Greater than 65 %
Plexiglass+**B**	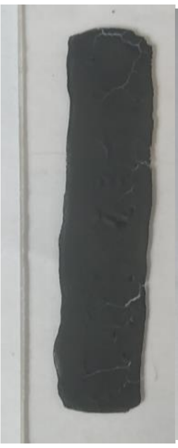	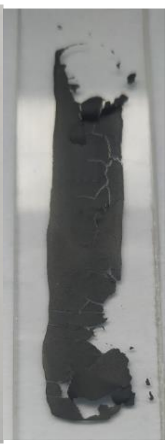	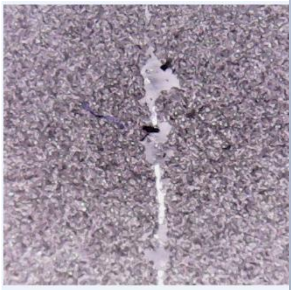	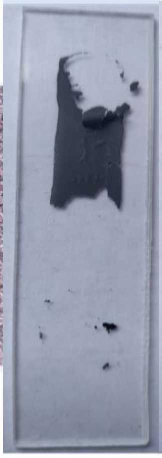	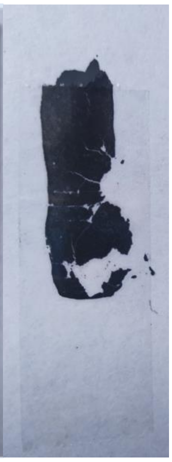	0B	Greater than 65 %
Plexiglass+**Base**	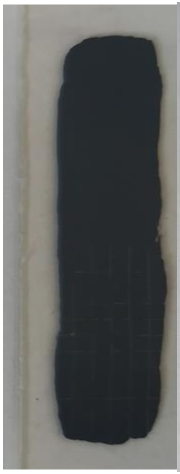	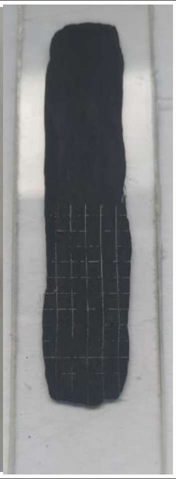	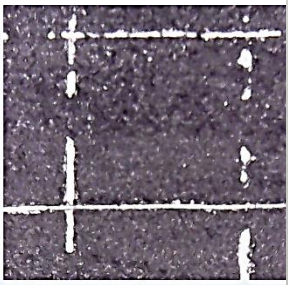	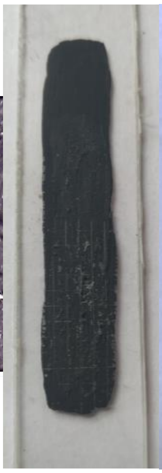	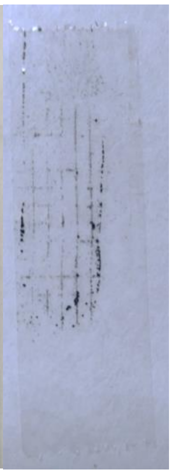	4B	Less than 5 %

PET + **A**	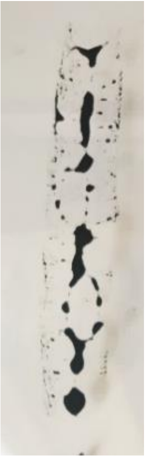	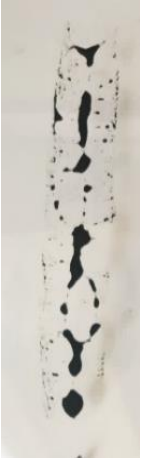	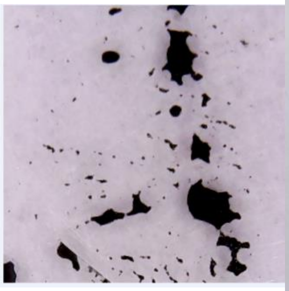	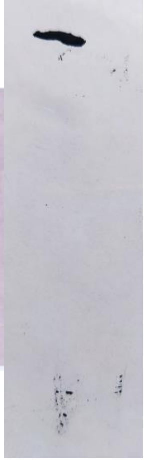	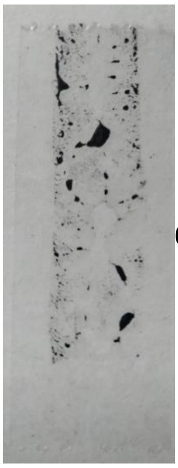	0B	Greater than 65 %
PET +**B**	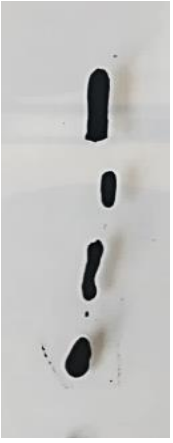	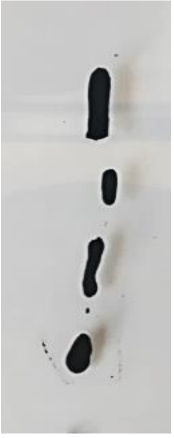		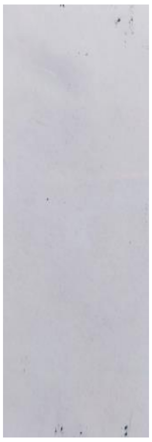	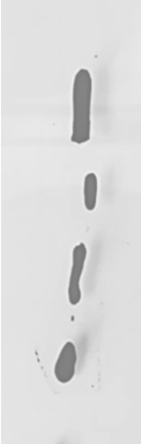	0B	Greater than 65 %
PET+**Base**	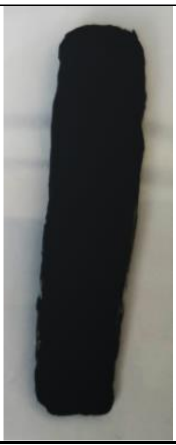	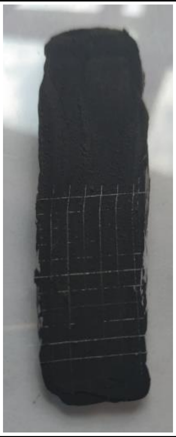	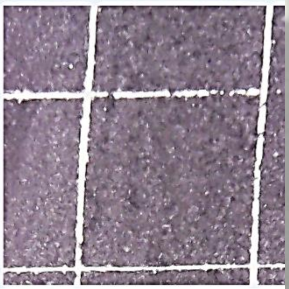	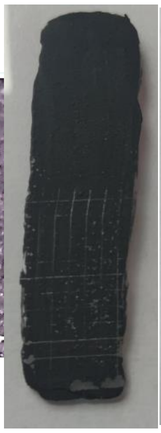	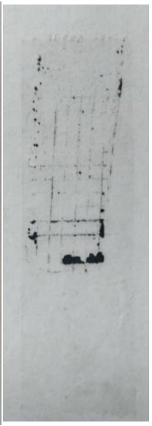	4B	Less than 5 %

PBT (the case of dosimeter+**A**	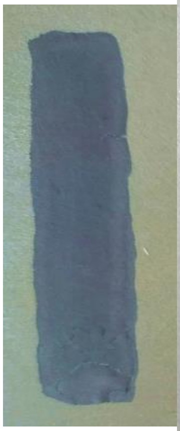	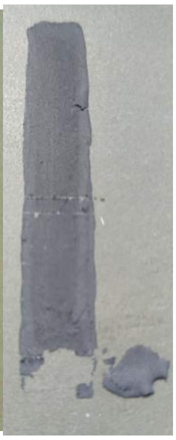	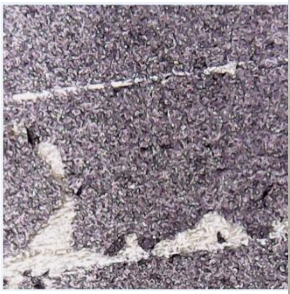	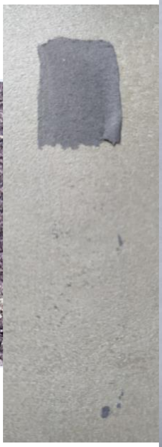	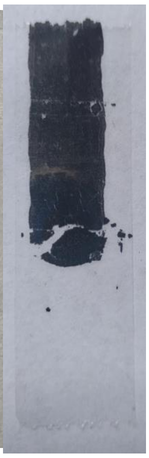	0B	Greater than 65 %
PBT (the case of dosimeter} + **B**	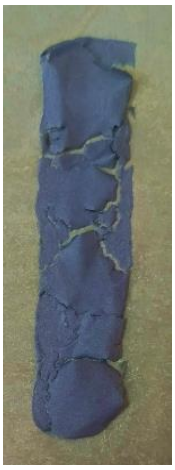	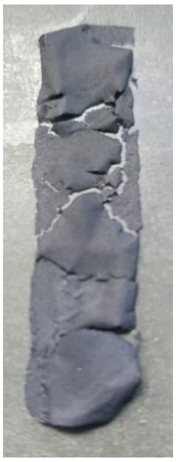	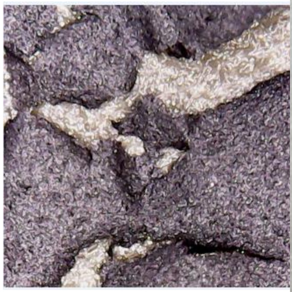	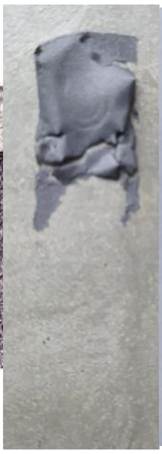	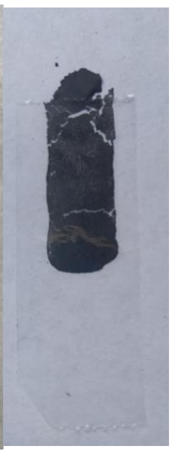	0B	Greater than 65 %
PBT (the case of dosimeter) + **Base**	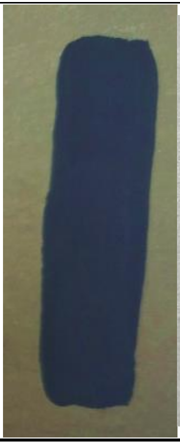	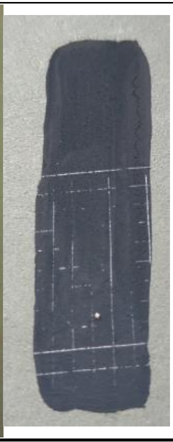	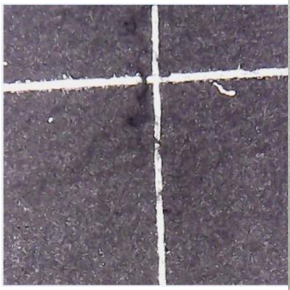	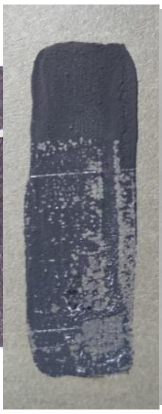	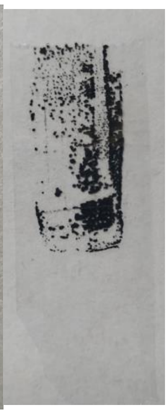	1B	35-65 %

Polyacetal (the case of termal imager) + A	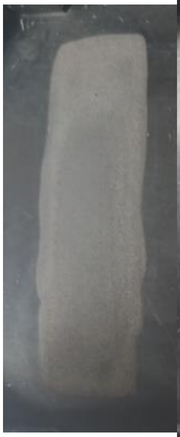	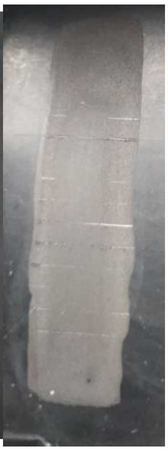	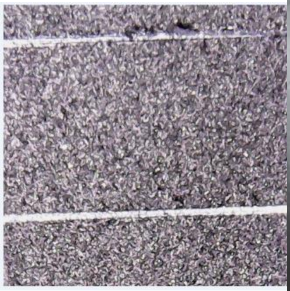	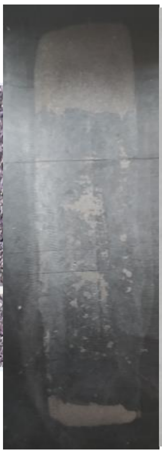	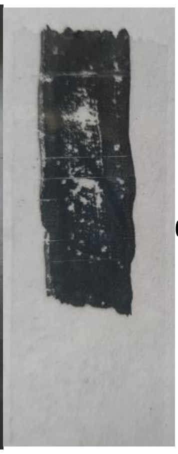	0B	Greater than 65 %
Polyacetal (the case of termal imager) + B	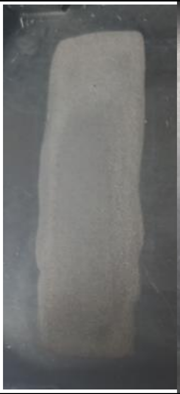	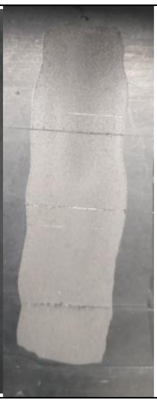	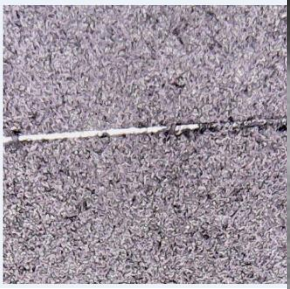	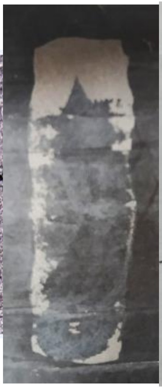	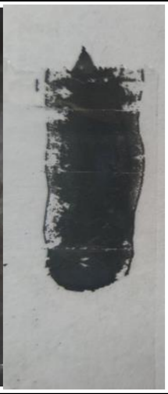	0B	Greater than 65 %
Polyacetal (the case of termal imager) + Base	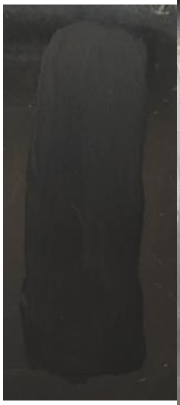	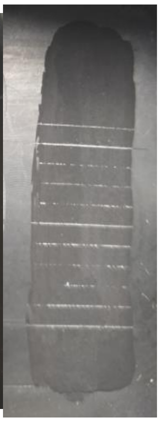	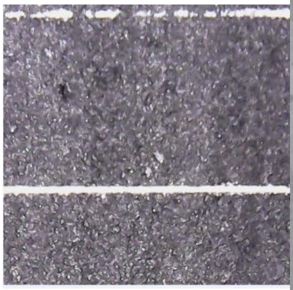	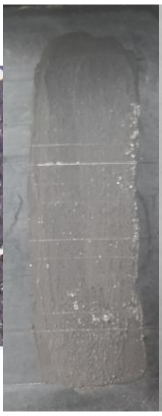	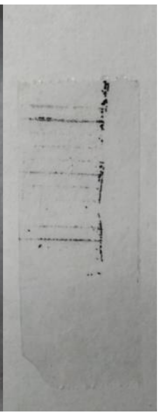	4B	Less than 5 %
Caprolone+**A**	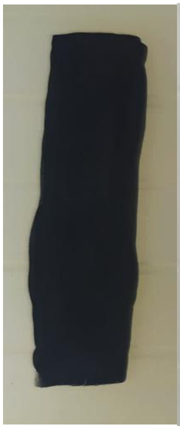	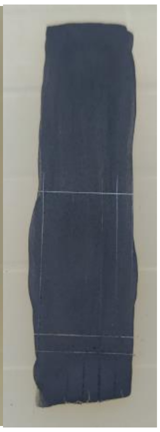	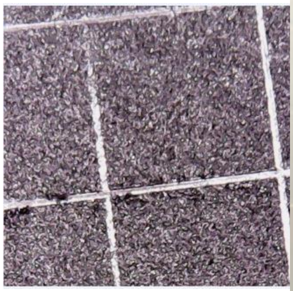	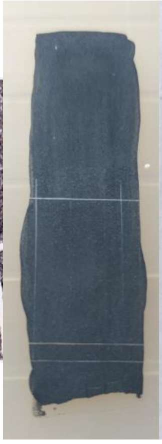	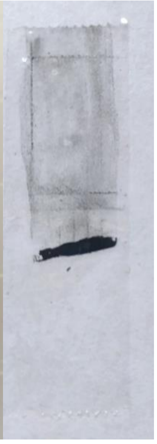	5B	0 % None

Caprolone + **B**	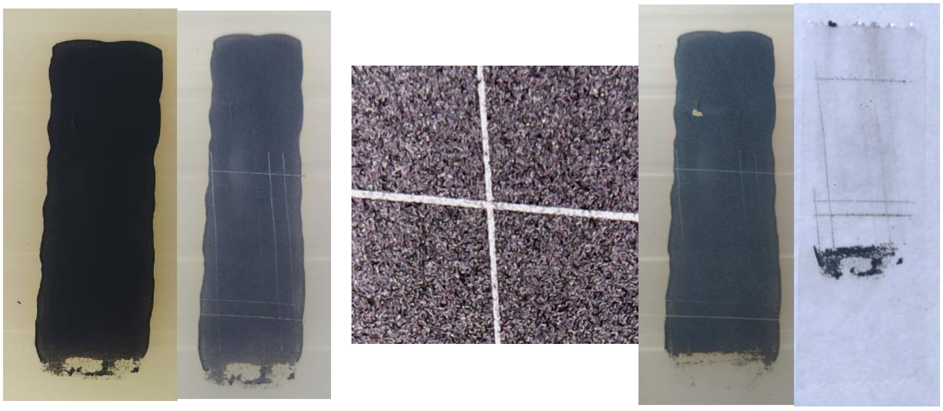	5B	0 %<br>None
Caprolone + **Base**	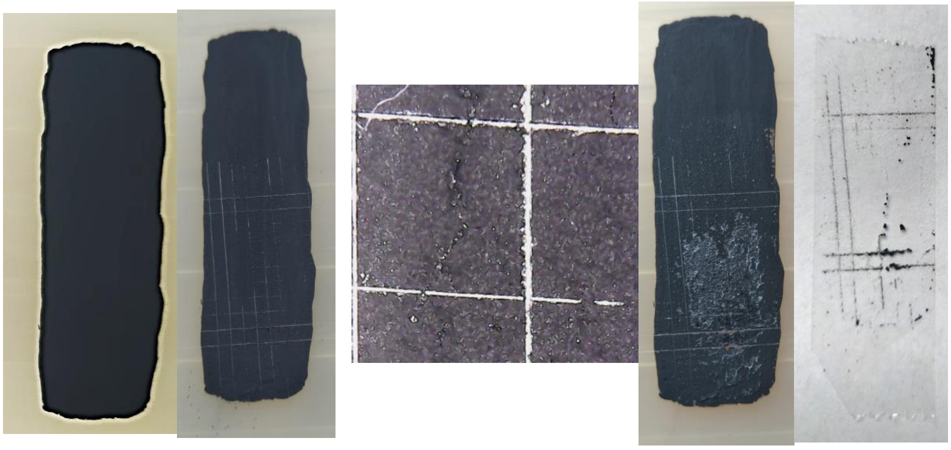	4-5B	0 %<br>None,<br>Less than<br>5 %
Concrete+**A**	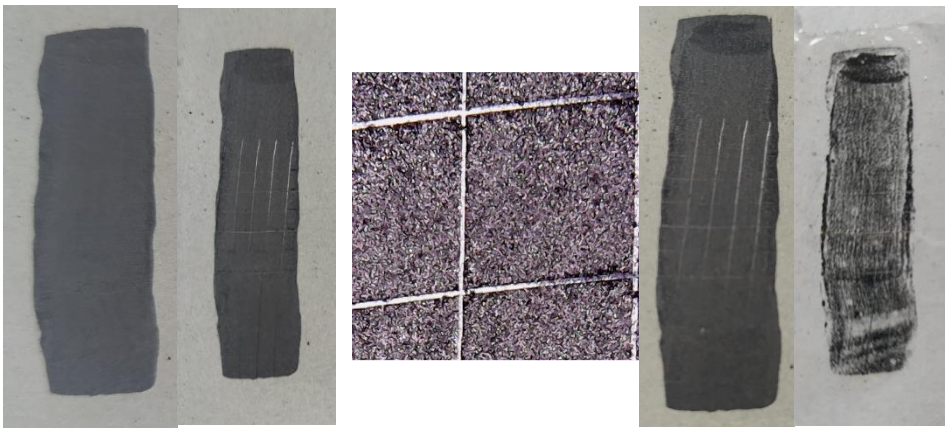	Exfoliate a thin top layer	
Concrete+**B**	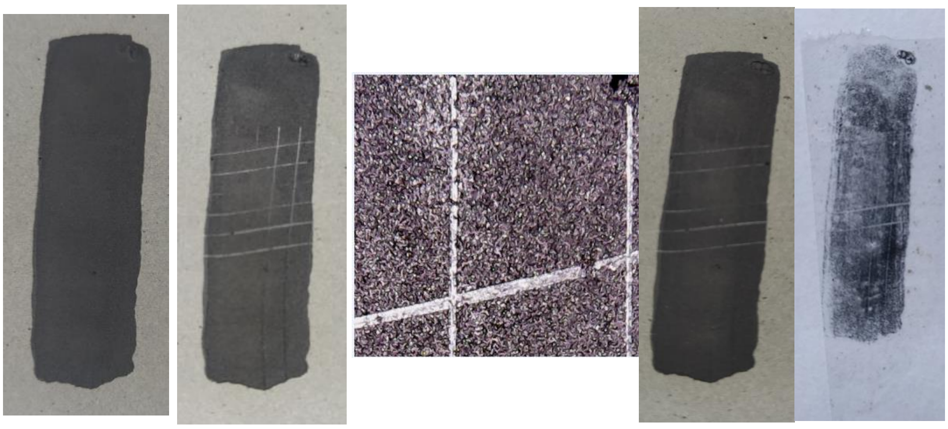	Exfoliate a thin top layer	

Concrete+ **Base**	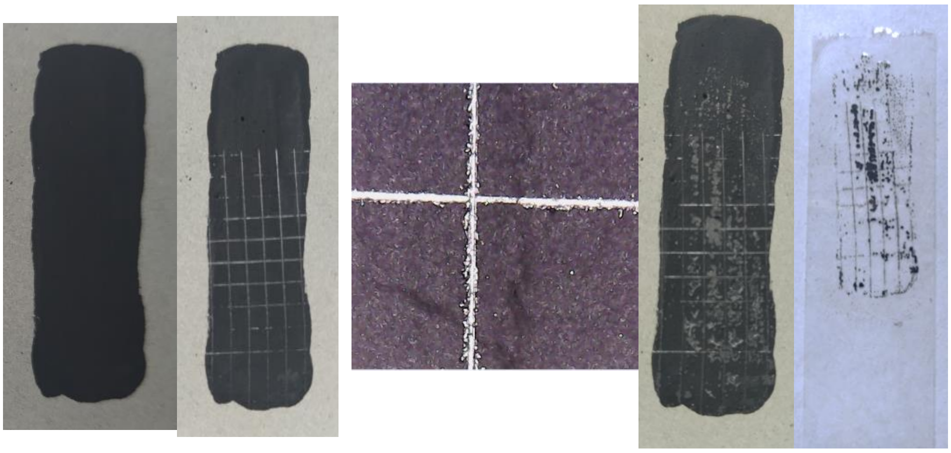	2B	15-35 %
Cardboard+**A**	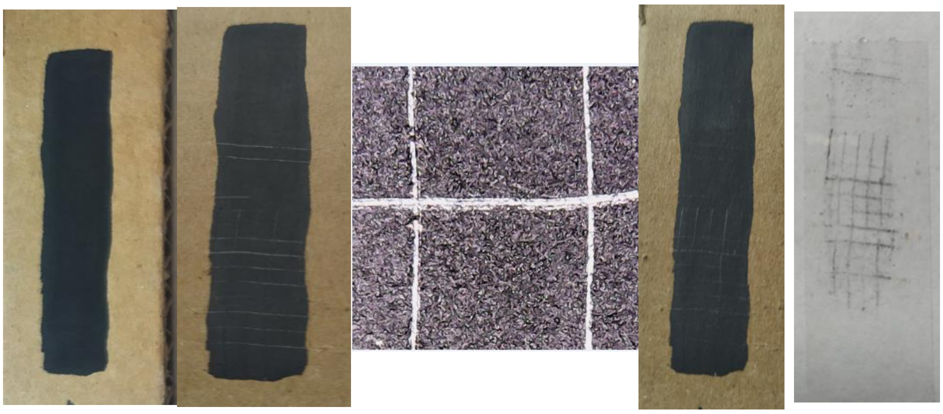	4-5B	0 %<br>None,<br>Less than<br>5 %
Cardboard+ **B**	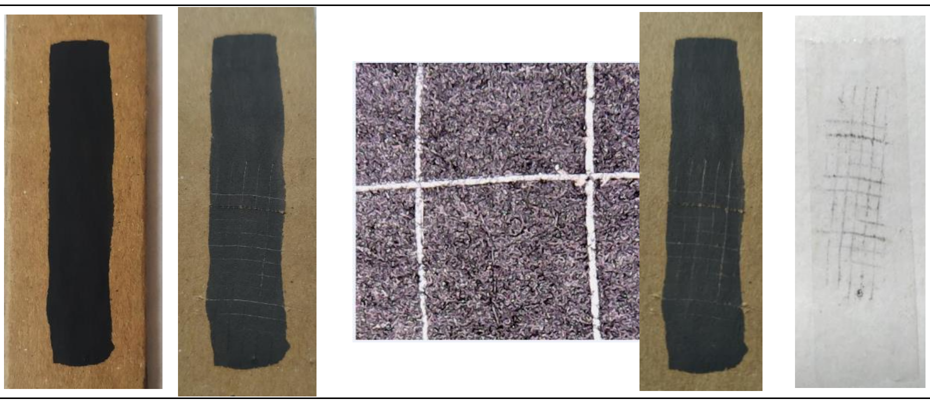	4-5B	0 %<br>None,<br>Less than<br>5 %
Cardboard+ **Base**	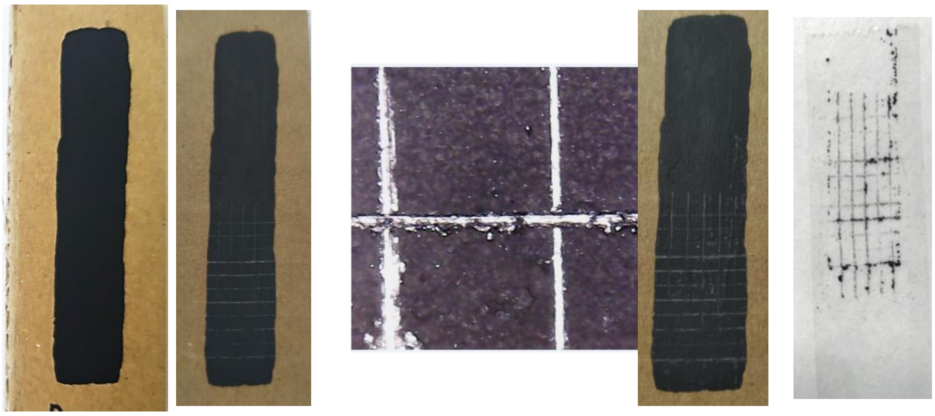	3B	5-15 %

Tile+**A**	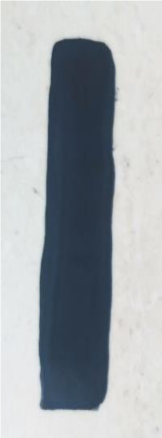	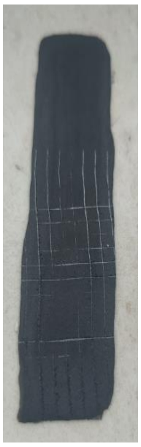	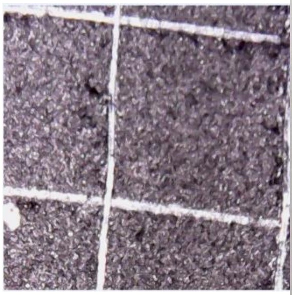	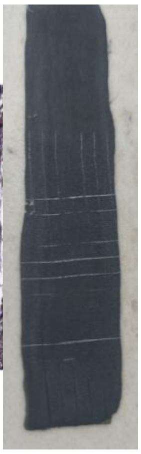	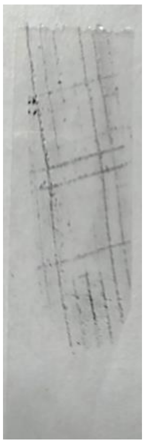	4B	Less than 5 %
Tile+ **B**	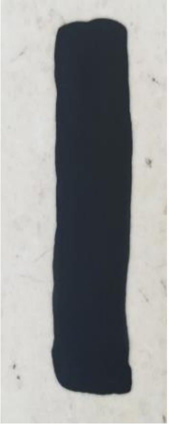	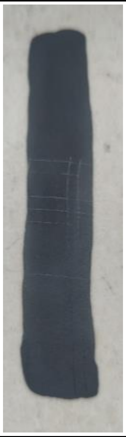	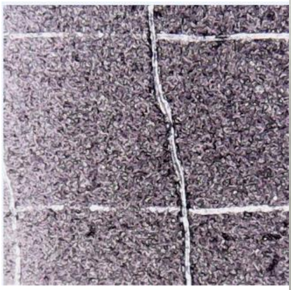	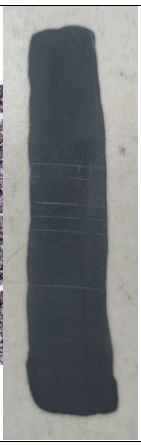	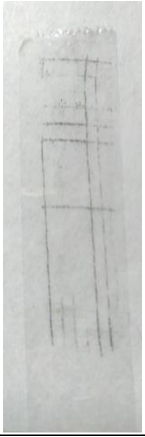	5B	0 % None
Tile+ **Base**	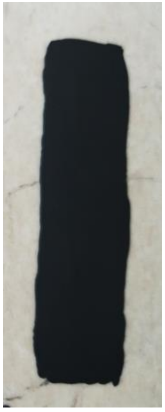	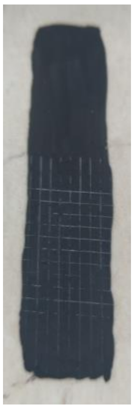	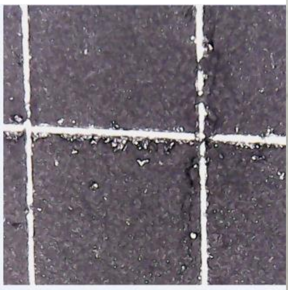	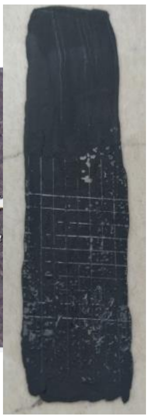	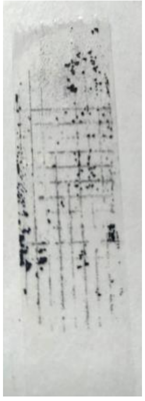	3B	5-15%

***Legend: Classification of Adhesion Test Results for Test Method.**

CLASSIFICATION OF ADHESION TEST RESULTS		
CLASSIFICATION	PERCENT AREA REMOVED	SURFACE OF CROSS-CUT AREA FROM WHICH FLAKING HAS OCCURRED FOR SIX PARALLEL CUTS AND ADHESION RANGE BY PERCENT
5B	0 % None	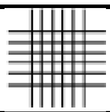
4B	Less than 5 %	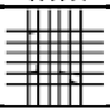

3B	5-15 %	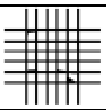 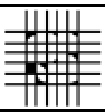
2B	15-35 %	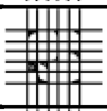 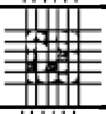
1B	35-65 %	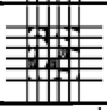 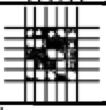
0B	Greater than 65 %	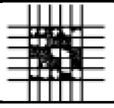
